# Growing Up Under Constant Light: A Challenge to the Endocrine Function of the Leydig Cells

**DOI:** 10.3389/fendo.2021.653602

**Published:** 2021-03-16

**Authors:** Dijana Z. Marinkovic, Marija L. J. Medar, Alisa P. Becin, Silvana A. Andric, Tatjana S. Kostic

**Affiliations:** Laboratory for Chronobiology and Aging, Laboratory for Reproductive Endocrinology and Signaling, Department for Biology and Ecology, Faculty of Sciences, University of Novi Sad, Novi Sad, Serbia

**Keywords:** Leydig cell, puberty, constant light, mitochondria, steroidogenesis, clock

## Abstract

The factors influencing Leydig cell maturity and the acquisition of functional capacity are incompletely defined. Here we analyzed the constant light (LL) influence on Leydig cells’ endocrine function during reproductive maturation. Rats were exposed to LL from P21 to P90. Data were collected at juvenile (P35), peri/pubertal (P42, P49), and adult (P90) stages of life. The results proved the effect of LL on rats’ physiology by changing of bimodal voluntary activity pattern into free-running. Additionally, the peripheral clock in Leydig cells changed in LL condition, indicating disturbed rhythm: the positive element (*Bmal1*) increased in pre-/pubertal but decreased in the adult period, while negative elements (*Per2* and *Reverba*) were increased. The effects of LL were most prominent in puberty: pituitary genes encoding gonadotropic hormones (*Cga, Lhb, Fshb*) decreased; serum corticosterone increased, while serum androgens and mass of testicular and sex accessory organs reduced; markers of Leydig cells maturity/differentiation (*Insl3*, *Lhcgr*) and steroidogenesis-related genes (*Scarb1, Star, Cyp11a1, Cyp17a1*) decreased; the steroidogenic and energetic capacity of the Leydig cell mitochondria decreased; the mtDNA copy number reduced, and mitochondrial dynamics markers changed: fusion decreased (*Opa1* and *Mfn2*), and mitophagy increased (*Pink1*). In adults, the negative effect of LL on mitochondrial function and steroidogenic capacity persists in adult Leydig cells while other parameters reached control values. Altogether, the results indicate that LL slows down Leydig cells’ maturation by reducing the endocrine and energy capacity of cells leading to the delay of reproductive development.

## Introduction

Most physiological processes in our body oscillate daily and are synchronized with external environmental changes (1). Light is the external environment element that profoundly influences circadian and neuroendocrine control of mammal physiology. According to the photoperiod, almost all hormones essential for life, including reproductive hormones, are secreted in a circadian rhythm. Adequate exposure to environmental cues such as the light/dark cycle is critical to the body physiology’s temporal organization. However, the urban lifestyle has reduced many external cues’ daily contrasts, including light/darkness contrasts. Many people are not exposed to a strong light/dark cycle spending most of their time indoors with the low light distinction between day and night (2), which can significantly disrupt rhythms, body physiology, and health. How living and growing up in buffered oscillations of external factors, including the light/dark cycle, affects males’ reproductive maturation remains to be clarified.

In males, testosterone is a crucial androgenic hormone that regulates fertility, development, and maintenance of the male reproductive system’s organs and muscle strength, cognition and sexual function, and overall male phenotype (3). In rat circulation, testosterone exhibits a low-amplitude daily rhythm with a peak at the beginning of the dark phase (4).

Leydig cells are the largest and most important source of androgenic hormones in mammals. In male life, two populations of Leydig cells exist. Fetal Leydig cells produce androgens and INSL3 in embryonic life crucial for the male fetus’s masculinization (5, 6). Androgenic activity of the fetal Leydig cells continues for a few weeks after birth, and after that, they disappear, followed by hormonal quiescence until puberty. When the hypothalamic–pituitary–testicular axis is reactivated, the new Leydig cells aroused from the testicular stem cells become able to produce testosterone. In rodents, in postnatal days from P21 to P35, the number of new progenitors Leydig cells increases together with differentiation, causing a rise in serum androgens (7). Those progenitor Leydig cells express genes related to steroidogenesis such are luteinizing hormone receptor (LHCGR), scavenger receptor Class B member 1 (SCARB1), steroidogenic acute regulatory protein (STAR), cytochrome P450 cholesterol side-chain cleavage (CYP11A1), 3*β*-hydroxysteroid dehydrogenase 1 (HSD3B1), and cytochrome P450 17*α*-hydroxylase/17,20-lyase (CYP17A1). However, those (progenitors) Leydig cells do not express 17*β*-hydroxysteroid dehydrogenase 3 (HSD17B3), the enzyme which synthesizes testosterone in the last step of the steroidogenic pathway. The activities of CYP11A1, HSD3B1, and CYP17A1 increase in immature Leydig cells as they mature from progenitor Leydig cells. The immature Leydig cells express HSD17B3, and therefore they can make testosterone from androstenedione. The main secretory product of these cells is not testosterone because they express steroid 5a-reductase 1 (SRD5A1) and 3a-hydroxysteroid hydrogenase (AKR1C14) therefore secrete androstanediol (3, 8, 9). Around P49–P56, the adult Leydig cells are formed in rat testis (7) characterized by higher expression of CYP11A1, HSD3B, CYP17A1, and HSD17B3 and silenced SRD5A1 expression (10, reviewed in Chen et al., 2020), which makes testosterone the primary secretory product of the cells. In general, the transitions along the Leydig cell lineage are associated with the progressive down-regulation of the proliferative activity, and the up-regulation of steroidogenic capacity. This is regulated by many signaling pathways specific for each step, including growth factors, such are Dessert Hedgehog, platelet-derived growth factor-AA, LH, and others (9). Another characteristic of adult Leydig cells is the presence of numerous mitochondria necessary for the initiation of steroidogenesis, namely, the cholesterol is transported into the mitochondria by StAR and other proteins of transduceosome as a consequence of the cAMP-PRKA signaling activation created in a series of events occurring after LH binding to its receptor (LHCGR) (11). In the mitochondria, cholesterol is converted into pregnenolone by CYP11A1, starting series of events culminating with testosterone production (12). However, steroidogenic and bioenergetic cell demands are closely linked to mitochondrial dynamics (13). In cells with stimulated steroidogenesis, a tubular mitochondrial network is dominant (13) enabled by the coordinated action of mitofusion genes and proteins, including *Mtn1/2* and *Opa1*. Also, Leydig cell steroidogenesis is facilitated by the inhibition of mitofission due to PRKA-dependent phosphorylation of DRP1 (14). Moreover, the LH-cAMP signaling is involved in the regulation of new mitochondria generation and mitochondrial fusion/fission coupled with increased steroidogenesis and energetic function (15). Still, studying the events regulating mitochondrial dynamic in developmental Leydig cells critical for establishing normal male fertility at puberty is missing.

During reproductive maturation, the dynamic interaction among genome, epigenome, and stochastic and environmental factors contributes to acquiring the full endocrine capacity of the Leydig cells. In this study, the effect of constant light (LL) on Leydig cells’ endocrine ability during the period of reproductive maturation was analyzed. The results indicate that LL slows Leydig cells’ maturation by reducing the endocrine and energy capacity of cells and delay in reproductive development.

## Material and Methods

### Chemicals

Medium 199 containing Earle’s salt and L-glutamine (M199), Dulbecco’s Modified Eagle Medium (DMEM-F12 medium), Tris-Ethylenediaminetetraacetic (EDTA), Bovine serum albumin (BSA), collagenase Type IA, from *Clostridium histolyticum*, Trypan Blue, tris(hydroxymethyl)aminomethane (Trizma base), and 4-(2-hydroxyethyl)-1-piperazineethanesulfonic acid (HEPES) were obtained from Sigma Chemical Company (St. Louis, MO, USA). TMRE (tetramethylrhodamine ethyl) was purchased from Fluka Company. hCG-Pregnyl 3,000 IU/mg (human chorionic gonadotropin) was from Organon (Roseland, New Jersey, USA). Power SYBR Green PCR Master Mix was purchased from Applied Biosystems (Thermo Fisher Scientific, Waltham, MA, USA), while qPCR primers were from Integrated DNA Technologies (Coralville, Iowa, United States). Anti-testosterone-11-BSA serum No. 250 was kindly supplied by Gordon D. Niswender (Colorado State University, Fort Collins, CO). Testosterone was from New England Nuclear (Brisel, Belgium). Active charcoal—Norit A—was obtained from Serva (Heidelberg, Germany).

### Animals

Experiments were carried out on male *Wistar* rats raised and bred in the animal facility of the Faculty of Sciences, University of Novi Sad. Rats were raised at a controlled temperature of 22 ± 2°C with free access to water and commercially balanced food. When rats were separated from the mother on the 21st day of age, they were divided into two groups. The first group was raised under the controlled light regime of 14 h light–10 h dark (LD, control group), while the second group was exposed to constant light conditions (LL, experimental group) until 35, 42, 49, and 90 postnatal days of life (P35, P42, P49, P90). At a certain age, groups were decapitated in the morning, *i.e.*, 1 h after the light turned on to the control group. All experiments were approved by the Local Ethical Committee on Animal Care and Use of the National Council for animal welfare and the National Law for Animal Welfare (No. 323-07-0-08975/2019-05) and in accordance with the National Institutes of Health Guide for the Care and Use of Laboratory Animals (NIH Publications number 80 23, revised 1996, seventh edition). All experiments and laboratory procedures are conducted in accordance to Laboratory biosecurity guidance, WHO, September 2006.

### Detection of an Animal’s Voluntary Activity

To analyze the voluntary rat’s activity, the P60 rats from both groups were placed in individual cages with a running wheel system. The system was set to record turns of the wheel that the animal made every 6 min. The rhythmic activity was monitored from P60 to P90. Based on the collected data, the actograms were formed as one of the standard ways to represent circadian rhythms. The graphical representation of the animal’s activity (actograms) was formed using R software (16).

### Serum Collection, Body, and Organ Weight Measurement

The animal’s body weight was measured. The trunk blood was collected. Individual serum samples were stored at −80°C until usage. The reproductive organs’ weights (testes, seminal vesicles, dorsal and ventral prostate) were respectively measured.

### Collection and Preparation of Purified Leydig Cells

Leydig cells were isolated according to the same protocol previously described by our research group (17, 18). Briefly, after isolation, testes were decapsulated and the main blood vessel removed. Testicular tissue was placed in 50 ml plastic tubes containing 0.25 mg/ml collagenase; 1.5%-BSA; 20 mM HEPES-M199 (two testes per tube). Cell isolation was continued by placing plastic tubes into a shaking-water bath (15 min/34°C/140 cycles/min). To stop enzymatic reaction, 45 ml of cold medium was added, and seminiferous tubules were separated during filtration through Mesh № 100 (Sigma, St. Louis, Missouri, USA). The remaining interstitial cell suspension was centrifuged (160 *× g* for 5 min) and resuspended in 8 ml/tube DMEM-F12 medium. To separate Leydig cells from the others, the resuspended cell mixture was moved to a Percoll gradient with different densities (1.080, 1.065, and 1.045 g/ml) and centrifuged 1,100 *× g* for 28 min (brake free). When separated, the Leydig cells were collected from specific gradient fragments (1.080/1.065 g/ml and 1.065/1.045 g/ml) washed in M199-0.1% BSA and centrifuged at 200 *× g*/5 min. Cell precipitate was resuspended in 5 ml DMEM/F12 and used for the experiment. According to HSD3B staining (19), the presence of Leydig cells in the culture was more than 90%. As for Trypan blue exclusion test, cell viability was greater than 95%. The controls to validate this purification method’s comprehensiveness were challenges of purified cells (although few of them) from the inter-layers with hCG (20). Briefly, the cells from the inter-layers (few of them) were collected following the described procedures and incubated with/without (10 ng/ml), but androgen production was not detected.

### *Ex Vivo* Experiments

Leydig cells’ primary culture was obtained by plating 3 × 10^6^ cells in a Petri dish (55 mm) and placed in CO_2_ incubator at 34°C to attach and recover for 3 h. After the recovering period, cell media were changed, and cells were treated with/without hCG (50 ng/ml) for 2 h. Cell media were collected and stored for androgen level determination, while cells were stored for further analysis.

### Hormone Level Measurement

Androgen concentration was determined by RIA in serum and cell medium samples (17). Antitestosterone serum number 250 used in this study showed 100% cross-reactivity with testosterone and dihydrotestosterone but recognized also other androgens. Samples were measured in duplicate (sensitivity: 6 pg/tube; intraassay coefficient of variation: 5–8%; interassay coefficient of variation: 7.5%). For serum corticosterone levels (21), all samples were measured in duplicate in one assay by the corticosterone EIA Kit (Cayman, Ann Arbor, MI, USA) with 30 pg/ml as the lowest standard significantly different from the blank.

### ATP Level and Mitochondrial Membrane Potential (Δ*Ψ*m) Determination

The ATP level was determined using the ATP Bioluminescence CLS II kit following the manual instruction (Roche Diagnostics, Indianapolis, USA) published previously by our group (15). Leydig cells (1 × 10^6^/tube) were resuspended in boiling water and Tris-EDTA (1:9), incubated in the water bath (100°C/3 min), centrifuged (900 *× g*/1 min), and the supernatant was used for ATP measurement while cell pellet was further used for Bradford method analysis. Sample/standard and Luciferase reagent were mixed 1:1, and luminescence was measured by the Biosystems/luminometer (Fluoroscan, Ascent, FL). For Δ*Ψ*m detection, as we described before (15, 22), Leydig cells were placed in 96 well-plates (1 × 10^5^ cells/well) and incubated with tetramethylrhodamine (TMRE) staining for 20 min/34°C/5%CO_2_. Fluorescence was measured on fluorimeter (Fluoroscan, Ascent, FL) on excitation wavelengths 485 and 550 nm, while emission wavelengths were 510 and 590 nm. Cells were washed with 0.1%BSA–PBS and stored for protein quantification by Bradford method.

### Genomic DNA Purification, Total RNA Isolation, and qRT-PCR Analysis

Genomic DNA from Leydig cells was purified by Wizard^®^ Genomic DNA Purification Kit (Promega, Medison, WI, USA), and total RNA from Leydig cells and pituitary glands was isolated using GenEluteTM Mammalian Total RNA Miniprep (Qiagen, Hilden, Germany) and RNeasy kit reagents (Sigma, St. Louis, Missouri, USA), following a protocol recommended by the manufacturer. RNA quality was measured and validated by BioSpec-nano (Shimadzu Biotech, Kyoto, Japan). Following DNase-I treatment (New England Biolabs, Ipswich, Massachusetts, USA), the first-strand cDNA was synthesized using the High Capacity kit for cDNA preparation and according to the manufacturer’s instructions (Thermo Fisher Scientific, Waltham, MA, USA). Negative controls consisting of non-reverse transcribed samples as well as positive controls were included in each set of reactions. Quantification of gene expression was performed by real-time PCR reaction with SYBR Green technology on Mastercycler RealPlex gradientS (Eppendorf) device. It was obtained in standard conditions (50°C/2 min, 95°C/10 min; 40 cycles, each 95°C/15 s, and then 60°C/1 min). The reaction was performed in the presence of 5 µl cDNA and specific primers (**Supplemental Table 1**; all primers were designed to flank the intron regions). Each sample was run in duplicate, and *Gapdh* was used as endogen control.

### Statistical Analysis

Statistical analysis was performed using GraphPad Prism 5. Experimental results T + DHT and TMRE are shown as mean value ± SEM individual variation. qPCR results are shown as mean RQ value ± SEM for each group, where all Ct values are obtained from one measurement. Results from each experiment were analyzed by Mann–Whitney non-parametric test. Parameters of rhythmic activity (p, MESOR, Amplitude, and Acrophase) were obtained by cosinor method using Cosinor software fitted to 24 h period (https://cosinor.online/app/cosinor.php).

## Results

In this study, growing up in LL condition and the consequences of male’s reproductive maturation were studied. Rats were reared in constant light from the mother’s separation (P21) to the adult stage (P90) and analyzed on P35, P42, P49, and P90. The specific ages are chosen because they illustrate Leydig cell maturation: in the testes from juvenile rats, P21–35, dominates proliferative progenitor cells; in the testes from peri/pubertal rats, P42–P49, due to reactivation of reproductive axis a transition from immature to adult Leydig cells occurs; testicles from P90 rats contain the mature adult Leydig cells.

### Growing Up Under Constant Light Changed Activity Patterns, Body, and Reproductive Organs’ Weight

**Figure 1:**
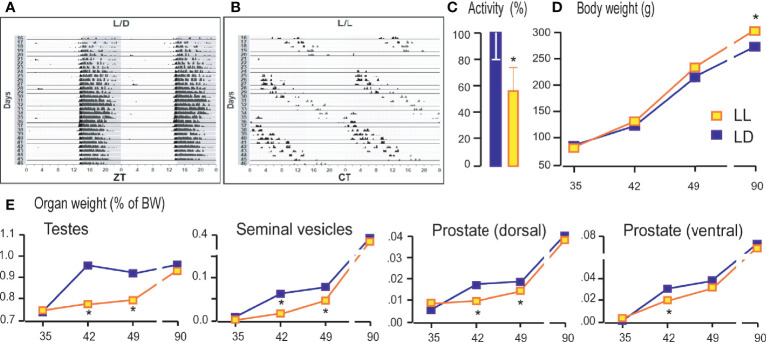
Growing up under constant light changed activity patterns, body and reproductive organs weight. Rats were raised under the controlled light regime of 14 h light–10 h dark (LD) or were exposed to constant light conditions (LL) until P35, P42, P49, and P90. From P60 to P90 the rats’ voluntary activity was monitored and actograms formed. The representative actograms from LD **(A)** and LL **(B)** condition as well as average daily activity **(C)** are shown. Rats were monitored for body mass **(D)** and weight of testes and accessory organs **(E)**. Data points/bars represent group means ± SEM values (n = 5–8). *Statistical significance at level p < 0.05 compared to the corresponding LD group.

Living under constant light changed the daily rhythms of the rat locomotor activity. The bimodal activity pattern detected in LD conditions (**Figure 1A**) became free-running so that the entire active period consistently drifted later each day (**Figure 1B**), and activity diminished slightly, leading to delay in acrophase (**Supplemental Table 2**). The period (*τ*) has been extended to 24.911 ± 0.07 h. Rats that grow up in constant light were 40% less active measured in the period from the sixtieth to the ninetieth postnatal day (**Figure 1C**). The body mass of rats growing up in constant light on days 35, 42, and 49 did not differ significantly from that of the controls. In both groups, the gradual increment of body mass was observed. However, in adulthood (P90), in the LL group an increase in body weight was observed compared to LD rats (**Figure 1D**). The weight of the testicles, seminal vesicles, and the ventral and dorsal prostate, which increased with age, were lower during puberty (42nd and 49th days) in rats that grew up in constant light (**Figure 1E**). Still, the weight of testes and sex accessory organs reach control values in adults (**Figure 1E**).

### Growing Up Under Constant Light Reduced Serum Androgens and Changed the Transcriptional Pattern of Genes Encoding Pituitary Gonadotropic Hormones

Decreased testicular mass and mass of sex accessory organs at puberty suggest that growing up in constant light affects the endocrine testicular function’s awakening. In that respect, serum androgens together with a transcription of genes encoding subunits of pituitary gonadotropins and the main marker of Leydig cell differentiation and activity were measured. All variables measured were compared with those observed in P35 rats that lived in LD conditions.

**Figure 2:**
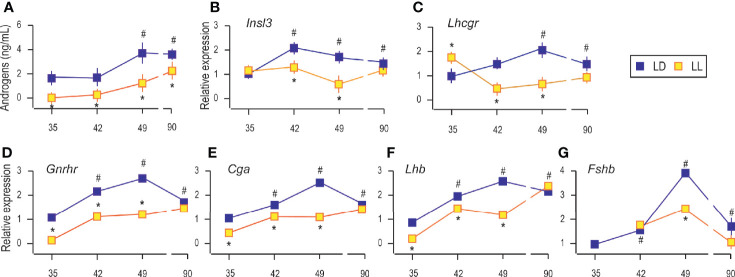
Growing up under constant light reduced serum androgens, changed transcriptional pattern of Leydig cells’ maturity markers and genes encoding pituitary gonadotropic hormones. Androgen levels were monitored in serum from rats that lived in LD or LL regime **(A)**. At a certain age Leydig cells were purified, RNA isolated, and qRT-PCR performed in order to measure expression of genes markers for Leydig cells’ maturity/activity **(B, C)**. The RNAs were isolated from the pituitary of both groups, and the expression of genes encoding GNRHR **(D)** as well as subunits of gonadotrophic hormones **(E–G)** was measured. Data points represent group means ± SEM values (n = 5–8). *Statistical significance at level p < 0.05 compared to the corresponding LD group;^#^Statistical significance at level p < 0.05 compared to the P35 LD group.

As it is well known, growing is accompanied by increased circulating androgens from the pubertal to the adult period (**Figure 2A**). Increased androgen production was followed by increased expression of *Insl3* (a marker of Leydig cell maturity and functionality; **Figure 2B**) and *Lhcgr* (gene encoding LH receptor, a marker of Leydig cells; **Figure 2C**) indicating the transition from immature to adult Leydig cells.

Rats raised in constant light had lower serum androgens than controls (**Figure 2A**), which may be associated with observed decreased testicular and accessory sex organ mass. Also, in LL conditions, Leydig cells derived from pubertal rats (P42 and P49) transcribed less *Insl3*, but transcript level approached the control values in adult rats (**Figure 2B**). Transcription of *Lhcgr* was decreased during puberty under constant light but came close to the values in Leydig cells from adults (**Figure 2C**). Since *Insl3* is a sensitive marker of Leydig cell maturity/function (23), the decreased androgens in circulation along with reduced *Insl3* and *Lhcgr* could reflect disturbance or delayed puberty due to life in constant light.

Since Leydig cell differentiation and androgenic activity are superiorly governed by hormones operated in the reproductive axis, the transcriptional pattern of genes encoding pituitary gonadotropic hormones (*Cga*, *Lhb*, and *Fshb*) as well as gonadotropin-releasing hormone receptor (*Gnrhr*) was studied. Results showed increased transcription of pituitary *Gnrhr*, *Cga*, and *Lhb* in P42, P49, and P90 compared to P35 (**Figures 2D–F**). Living in constant light reduced gene expression in P35, P42, and P49 but was equated with control values in Leydig cells from adult rats (**Figures 2D–F**). Also, *Fshb* increased with age. The highest expression was detected in pubertal P49 (**Figure 2G**). LL reduced *Fshb* in P49, while in Leydig cells from adults, transcription reached control values (**Figure 2G**).

### Growing Up Under Constant Light Changed the Transcriptional Pattern of Steroidogenesis-Related Genes

Further, the mRNA abundance of elements essential for steroidogenesis was analyzed in Leydig cells isolated from P35, P42, P49, and P90 rats that grow up in LD or LL conditions.

**Figure 3:**
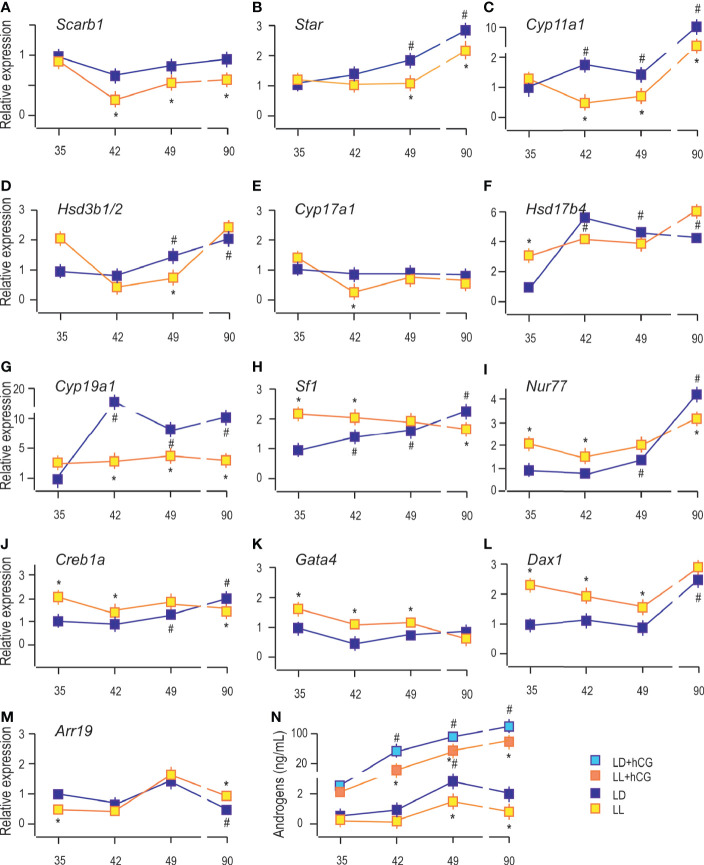
Growing up under constant light changed transcriptional pattern of steroidogenesis-related genes. RNAs were isolated from purified Leydig cells and transcription of steroidogenic genes was monitored **(A–G)** together with transcription of positive **(H–K)** and negative **(L, M)** steroidogenic regulators. Leydig cells obtained from different LD and LL groups were challenged with/wo hCG (bind to LHR; 50 ng/ml) followed by androgen levels determination **(N)**. Data points represent group means ± SEM values (n = 5). *Statistical significance at level p < 0.05 compared to the corresponding LD group;^#^Statistical significance at level p < 0.05 compared to the P35 LD group.

The analysis showed an age-associated increment of transcription of some Leydig cell biomarkers, including steroidogenic acute regulatory protein (*Star*), enabled cholesterol transport into the mitochondria and a set of androgen synthases, such as cytochrome P450 cholesterol side-chain cleavage (*Cyp11a1*), 3*β*-hydroxysteroid dehydrogenase 1/2 (*Hsd3b1/2*), cytochrome P450 17*α*-hydroxylase/17,20-lyase (*Cyp17a1*), 17*β*-hydroxysteroid dehydrogenase (*Hsd17b4*) and an androgen metabolizing enzymes, such as aromatase (*Cyp19a1*) (9). Living in constant light decreased transcripts of genes essential for androgen production: scavenger receptor Class B member 1 (*Sarb1*; involved in cholesterol delivery into cells) in Leydig cells from P42, P49, and P90 rats (**Figure 3A**); *Star* in P49 and P90 (**Figure 3B**); *Cyp11a1* in P42 to P90 (**Figure 3C**); *Hsd3b1/2* in P49 (**Figure 3D**); *Cyp17a1* in P42 (**Figure 3E**), and *Cyp19a1* in Leydig cells from P42 to P90 (**Figure 3G**). The *Hsd17b4* transcription was increased in P35 under the influence of constant light but without changes in other investigated age categories (**Figure 3F**).

Further, the qRT-PCR analysis revealed increased abundance of steroidogenic stimulators, *Sf1, Nur77*, and *Creb1a* (**Figures 3H–J**), as well as a steroidogenic repressor, *Dax1* (**Figure 3L**). In contrast, *Arr19*, a repressor of steroidogenic genes, decreased (**Figure 3M**) in Leydig cells from the adult testis. We could not detect significant changes in the transcriptional pattern of *Gata4* during pubertal maturation (**Figure 3K**). The LL condition stimulated *Sf1, Nur77*, *Creb1a, Gata4,* and *Dax1* in Leydig cells from P35 to P49 rats (**Figures 3H–L**). Still, in P90 Leydig cells, the steroidogenic stimulators (*Sf1, Nur77*, and *Creb1a*) were decreased (**Figures 3H–J**) and repressor, *Arr19*, increased (**Figure 3M**), supporting the observed attenuated transcription of steroidogenic genes. LL did not influence *Gata4* and *Dax1* mRNA levels (**Figures 3K, L**).

Further, to elucidate the resulting differences in maturity observed by the changed expression of *Lhcgr* and steroidogenic genes, Leydig cells obtained from P35 to P90 rats that lived in LD or LL regime were challenged with hCG. Leydig cells derived from rats that lived in the LL regime exhibited a reduced response to hCG stimulation compared to control LD rats (**Figure 3N**). This was illustrated by the reduced increase of androgen production (**Figure 3N**) and suggested the attenuated steroidogenic machinery level.

### Constant Light Changed Leydig Cell’s Mitochondrial Function

**Figure 4:**
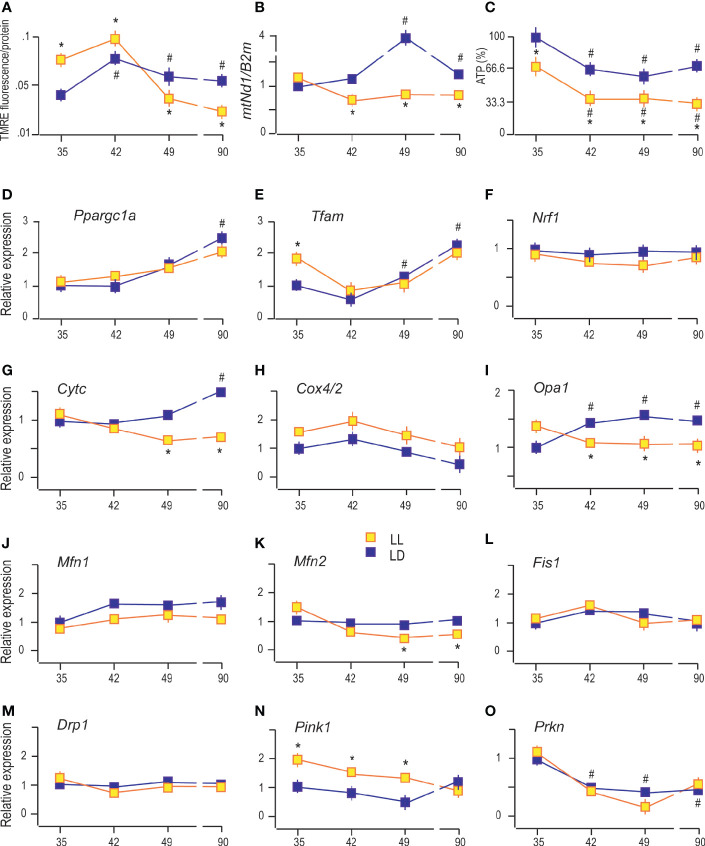
Constant light changed Leydig cell’s mitochondrial function. The Δ*ψ*m was measured by using TMRE fluorescence **(A)**, followed by monitoring of mtDNA content estimated through *mtNd1/B2m* ratio **(B)** and analysis of ATP levels **(C)** in purified Leydig cells isolated from rats of different ages of both (LD and LL) groups. Also, transcriptional patterns of genes important for mitochondrial biogenesis **(D–F)**, genes encoding subunits of respiratory proteins **(G, H)**, mitochondrial fusion **(I–K)**, mitochondrial fission **(L, M)** and mitophagy **(N, O)** were monitored in the same cells. Data points represent group means ± SEM values (n = 5). *Statistical significance at level p < 0.05 compared to the corresponding LD group; ^#^Statistical significance at level p < 0.05 compared to the P35 LD group.

In Leydig cells, the steroid production begins in the mitochondria enabled by cholesterol availability and mitochondrial steroidogenic enzyme localization. For efficient steroid production, functional mitochondria are necessary (24), so some characteristics which provide insight into mitochondrial functionality were analyzed. Since mitochondrial membrane potential (Δ*ψ*m) contributes to mitochondrial energetic and steroidogenesis (12), it was essential to analyze possible changes of Δ*ψ*m in Leydig cells during puberty in LL conditions. Changes in the Δ*ψ*m was detected by measuring TMRE fluorescence because TMRE fluorescence values are proportional to the magnitude of Δ*ψ*m. The obtained results showed increased Δ*ψ*m in Leydig cells during puberty and adulthood (**Figure 4A**), followed by an increase in the number of mtDNA copies estimated through *mtNd1/B2m* ratio (**Figure 4B**). Both data indicate the increased mitochondrial engagement needed for enlarged steroid production. Moreover, a positive correlation was observed between serum androgens and Leydig cells’ mtDNA content (R = 0.801). However, life in LL conditions increased Δ*ψ*m at the beginning of the pubertal period (P35 and P42) but was followed by subsequent reduction (P49 and P90) (**Figure 4A**). Additionally, LL caused a decrease in mitochondrial DNA content in the pubertal period and adulthood (**Figure 4B**).

On the other side, ATP production in Leydig cells gradually decreased with rats’ ages (**Figure 4C**). Even more, LL reduced the production of ATP in Leydig cells from all four age categories that have been studied (**Figure 4C**). Decreased ATP, mtDNA, and Δ*ψ*m together with decreased steroid production support the hypothesis that growing up in LL suppresses mitochondrial function in Leydig cells.

It is well known that mitochondrial function is closely linked with fusion/fission (25), while mitochondrial mass is regulated by mitochondrial biogenesis and mitophagy (26). To see if puberty and growing under LL are accompanied by altered mitochondrial biogenesis or mitophagy and/or by changes in mitochondrial fusion/fission, the expression of genes involved in these processes was monitored.

The obtained results indicate gradually increased expression of *Ppargc1a* (main regulator of mitochondrial biogenesis and function) in Leydig cells from pubertal and adult rats compared with cells from prepubertal rats (**Figure 4D**). The same expression pattern showed its downstream gene *Tfam* (**Figure 4E**) and *Cytc* (**Figure 4G**), indicating increased expression of the markers of mitochondrial biogenesis. However, the expressions of *Nrf1* (activator of genes required for respiration) and *Cox4/2* (encoding subunit of respiratory protein) were not changed during sexual maturation (**Figures 4F, H** respectively). LL did not significantly change the transcriptional pattern of *Ppargc1a, Tfam, Nrf1, and Cox4/2* but decreased *Cytc* in P49 and P90 (**Figures 4D, E, F, H, G**, respectively).

Reproductive maturation in Leydig cells increased transcription of the main regulator of mitochondrial crista architecture and profusion gene, *Opa1* (**Figure 4I**), without effect on other profusion genes, *Mfn1* (**Figure 4J**) and *Mfn2* (**Figure 4K**). The profission *Fiss1* (**Figure 4L**) and *Drp1* (**Figure 4M**) were not changed during Leydig cell maturation. However, the LL regime changed the mitochondrial dynamics by reducing *Opa1* and *Mfn2* expression during puberty and adulthood without effect on *Mfn1*, *Fiss1*, and *Drp1* (**Figures 4I–M**).

Further, sexual maturation has affected Leydig cells’ mitophagy by reducing transcription of *Prkn* (**Figure 4O**) without effect on *Pink1* (**Figure 4N**). However, life under LL conditions stimulated *Pink1* (**Figure 4N**) in Leydig cells from P35 and P42 and P49 rats. The obtained results suggest that growing up under LL conditions stimulates mitophagy and inhibits mitochondrial biogenesis in Leydig cells leading to decreased mitochondrial mass.

### Constant Light Changed the Expression of Clock Genes in Leydig Cells

**Figure 5:**
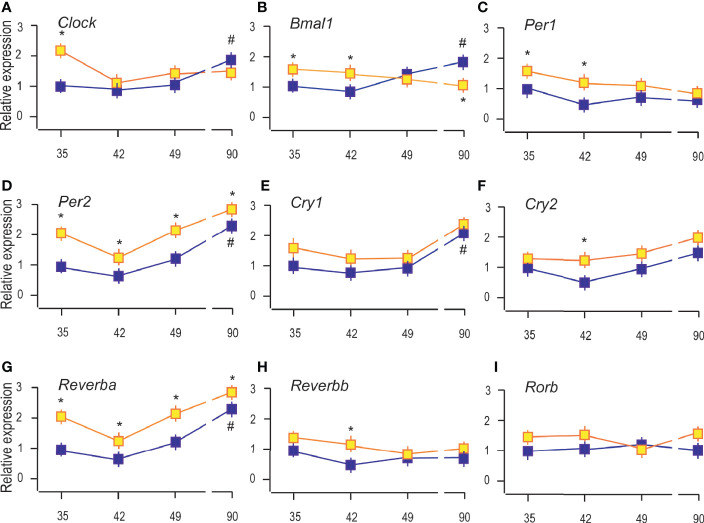
Constant light changed the expression of clock genes in Leydig cells. RNAs were isolated from Leydig cells obtained from rats of different ages of both (LD and LL) groups and transcription of clock genes were estimated by qRT-PCR. The transcription of positive clock elements **(A, B)** and negative elements **(C–F)** from primary clock loop together with elements from the secondary loop **(G–I)** was shown. Data points represent group means ± SEM values (n = 5). *Statistical significance at level p < 0.05 compared to the corresponding LD group; ^#^Statistical significance at level p < 0.05 compared to the P35 LD group.

Leydig cells are known to have a rhythmic endocrine function in addition to the rhythmic expression of clock genes (4, 27). To estimate the effect of LL on the Leydig cells’ clock, qRT-PCR analysis of the canonical clock gene expression was done (**Figure 5**). Clock genes’ expression was estimated 1 h after lights were turned on in the LD group. The gene expression in Leydig cells from P42, P49, and P90 was compared with P35 rats.

The results showed that reproductive maturation increased transcription of clock positive elements (*Clock* and *Bmal1*; **Figures 5A, B**) but also of negative elements (*Per2*, *Cry1*, and *Reverba*) (**Figures 5D, E, G**), suggesting that clockwork in Leydig cells depends on cell maturation. However, *Per1*, *Cry2*, *Reverbb*, and *Rorb* were not significantly changed during Leydig cell maturation (**Figures 5C, F, H, I**). The LL increased the expression of *Clock* and *Bmal1* in the Leydig cells from P35 and P42, but *Bmal1* was reduced in adult Leydig cells from P90 rats (**Figures 5A, B**). Transcriptional level of *Per2* and *Reverba* was increased in all the investigated age-categories (**Figures 5D, G**) while *Cry2* and *Reverbb* increased in Leydig cells from pubertal P42 rats (**Figures 5F, H**). The results indicate the stimulatory effect of LL on clock repressive elements *Per2*, *Cry1*, and *Reverba* in immature and adult Leydig cells and inhibitory on positive *Bmal1* in adult Leydig cells and propose a disruption of the clock and endocrine rhythm in Leydig cells.

### Growing Up Under Constant Light Increased Blood Corticosterone and Changed the Transcriptional Pattern of Glucocorticoid-Signaling Elements

**Figure 6:**
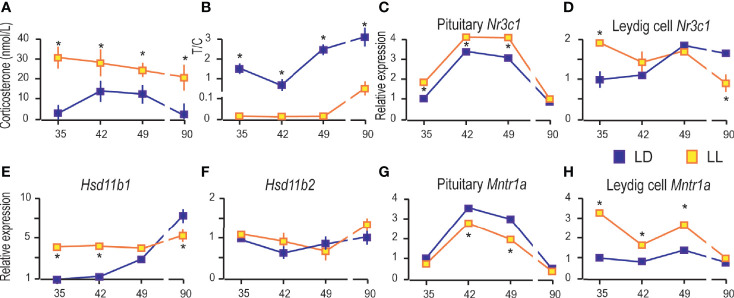
Growing up under constant light increased blood corticosterone and changed the transcriptional pattern of glucocorticoid-signaling elements. Corticosterone levels were monitored in serum from rats that lived in LD or LL regime **(A)**. The blood testosterone/corticosterone (T/C) ratio is presented during growing up **(B)**. At a certain age Leydig cells were purified, RNA isolated, and qRT-PCR performed in order to measure expression of genes involved in corticosterone signaling: pituitary *Nr3c1* **(C)**, Leydig cells’ *Nr3c1* **(D)** and genes encoded local regulators of glucocorticoid levels **(E, F)**. Relative mRNA expression of pituitary **(G)** and Leydig cells *Mntr1a* **(H)**. Data points represent group means ± SEM values (n = 5–8). *Statistical significance at level p < 0.05 compared to the corresponding LD group.

Since living in constant light could activate chronic stress-response, the blood corticosterone was measured. The results revealed increased corticosterone levels in the blood of rats which lived under continuous lighting (**Figure 6A**). The ratio between testosterone and corticosterone (T/C) in LL decreased (**Figure 6B**) suggesting possible connection with registered decreased activity (**Figure 1C**). Also, the results indicated higher T/C in the course of growth in both groups (**Figure 6B**).

Further, since corticosterone action is mainly mediated by the glucocorticoid receptor (GR), the transcription of gene-encoded GR (*Nr3c1*) was monitored in the pituitary and Leydig cells. Pituitary Nr3c1 was increased in P35–P49 under constant light, while in P90 the difference in respect to controls was not observed (**Figure 6C**). The Leydig cells’ *Nr3c1* was increased in P35 and decreased in P90 (**Figure 6D**).

Nevertheless, the dehydrogenase activity of enzymes 11BHSD1/2 has been showing to protect Leydig cells from the harmful effects of excessive glucocorticoid exposures (28, 29). In that respect, transcription analysis showed increased *Hsd11b1* in Leydig cells during rat growing up, so that it reached the highest level in P90 (**Figure 6E**). LL increased *Hsd11b1* in P35 and P42, but it was decreased in P90 (**Figure 6E**). The *Hsd11b2* transcription did not change during the course of growing up and living in LL conditions (**Figure 6F**).

Finally, we examined the effects of LL on the expression of genes encoding MNTR1A in the pituitary and Leydig cells. Our previous work showed that the *Mntr1a* and *Mntr1b* were transcribed in the hypothalamus and pituitary, although *Mntr1b* is less prevalent. In adult Leydig cells, transcription of *Mntr1a* was at the level of significance, although low transcription of *Mntr1a/b* was detected in testicular tissue (4). Living in LL decreased *Mntr1a* in the pituitary in the peripubertal period (P42 and P49) (**Figure 6G**), while in Leydig cells, it was increased in P35, P42, and P49 (**Figure 6H**).

## Discussion

It is enthroned knowledge that temporal organization is essential for maintaining good body physiology and health (1). The circadian system needs to be reset every day by environmental cues such as light/dark cycle, temperature changes, or food availability to synchronize body function with habitat conditions. Without external signals, in constant conditions, the circadian system will oscillate with its endogenous period, the so-called free-running period (1). Herein the consequences of growing up in constant conditions, *i.e.*, without temporal cues, on the development of Leydig cells’ endocrine capacity were studied. Our results indicate delayed maturation of Leydig cells in free-running conditions. The effect is most pronounced during puberty, although the consequences are also observed in adulthood.

**Figure 7:**
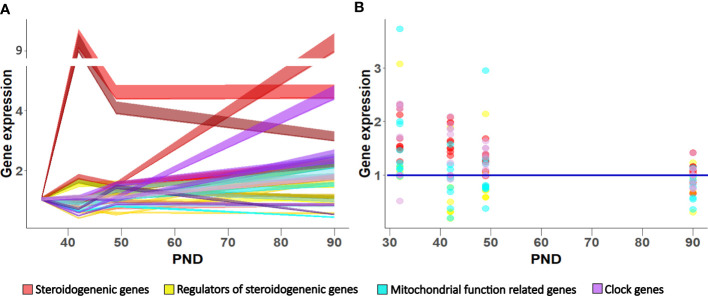
Gene expression pattern in Leydig cells during maturation in LD **(A)** and LL condition **(B)**. Data shown represent expression pattern of genes important for steroidogenesis, mitochondrial function and clock during Leydig cell maturation **(A)** relative to P35. Ribbons represent 95% confidence intervals. Effect of growing up in constant light regime on Leydig cell gene expression **(B)**. Points represent a deviation in the expression of a particular gene in LL condition in respect to corresponding LD (control value = 1; blue line).

Reproduction is essential for the survival and perpetuation of species. Hormones of the neuroendocrine regulatory circuit govern reproductive development with hierarchical cascades of regulatory feedback loops (30). In male reproductive development, puberty is a critical period that entirely depends on Leydig cells’ steroidogenic capacity increased by the awakening of the reproductive axis. Increased gradual expression of *Lhcgr* sensitizes cells’ to LH to recruit steroidogenic regulators, whose expression is also growing during puberty (*Creb1a*, *Sf1, Nur77*), to stimulate steroidogenic genes (*Star, Cyp11a1, Hsd3b1/2, Hsd17b4*). On the other side, repression of steroidogenic genes decline, associated with *Arr19* lowering. Besides steroids, the Leydig cell lineage transitions are also associated with increased *Insl3*, a critical biomarker reflecting Leydig cell functional capacity (23). Additionally, the enlarged transcription of clock genes in the adult Leydig cells points to the regulatory role of reproductive axis hormones on the Leydig cells’ circadian clock (31). Altogether, the Leydig cells’ maturation increases the steroidogenesis (**Figure 7A**) and the level of androgens in the circulation, enabling the development of sex accessory organs and male phenotype.

It is known that exposure to constant light causes rhythmic clock activity changes in suprachiasmatic neurons and subsequently peripheral clocks in different body cells (32–34). The suprachiasmatic neurons sense the environmental light through the retinohypothalamic pathway and send information to peripheral clocks, including the Leydig cell clock, through many hormones such as LH and melatonin (4, 31). However, an integrated network of signals linking the suprachiasmatic neurons and peripheral oscillators, including Leydig cells, is presently not well understood. This study confirms that growing up in constant environmental conditions changed body rhythm, activated free-run activity model with period longer than 24 h, and altered the transcriptional pattern of Leydig cell’s clock genes. In adult Leydig cells from rats growing up in LL, positive clock element *Bmal1* was down-regulated. Still, negative regulators, *Per2* and *Reverba*, were up-regulated, suggesting different clockwork in constant conditions and possibly changing the transcriptional pattern of clock-regulated genes. It is shown that BMAL1 (35) or REVERBa (36) could regulate the transcription of *Star* in steroidogenic cells.

In free-running conditions, the inhibition of pituitary *Gnrhr* and genes encoding gonadotropic hormones (*Cga, Lhb, Fshb*) in juvenile and peripubertal pituitary was detected. Accordingly, decreased serum LH was observed after prolonged exposure of rats to LL conditions (37). The observed reduction of pituitary *Cga/Lhb* is associated with decreased transcription of essential steroidogenic-related genes including *Scarb1*, *Star*, *Cyp11a1*, *Hsd3b*, all connected with cholesterol metabolism in the first steps of steroidogenesis (12) mostly operated in the mitochondria. Indeed, our results supported the significant role of the mitochondria in Leydig cell maturation.

However, despite the importance of the mitochondria in cellular and metabolic health, including steroidogenesis, the details about their organization and synchronization, especially during Leydig cells development, are not well characterized. Our results indicate increased mitochondrial biogenesis during Leydig cell maturation illustrated by increased primary mitochondrial biogenesis markers (*Ppargc1a, Tfam, Cytc*), namely, the *Ppargc1a*/PGC1a plays a crucial role in mitochondrial biogenesis through transcriptional regulation of its downstream genes such as *Nrf1*, *Nrf2*, and *Tfam*, leading to the synthesis of mitochondrial DNA, proteins, and generation of new mitochondria (38). Indeed, at puberty, the enlarged mtDNA content was observed coupled with increased mitochondrial membrane potential and likely increased fusion needed for efficient import necessary for steroidogenesis. However, ATP level in Leydig cells did not follow this upward trend.

On the contrary, the transitions along the Leydig cell lineage are associated with decreased cell energy capacity. Since for efficient steroidogenesis high polarized mitochondrial membrane is needed (24), results of increased steroidogenesis and decreased ATP production suggests dissociation of energetic and steroidogenic mitochondrial function. Progenitor Leydig cells are proliferative (9), having high metabolic energy requirements; with maturation, the number of divisions decreases, so the adult cells no longer divide, consequently associated with lower energy demand.

The mitochondrial fusion and fission regulate the formation of the mitochondrial network responsible for energetic and steroidogenic mitochondrial function (39). Both processes are enabled by the activity of several essential genes and their products, such are *Mfn1*/MFN1, *Mfn2*/MFN2, *Opa1*/OPA1, *Drp1*/DRP1, and *Fis1*/FIS1 (25). Mitofusion is a crucial step that allows transport of intermediate products in/out mitochondria and is essential for cholesterol import into mitochondria and steroid formation (13, 14, 40). When the reproductive axis wakes up or is active, in Leydig cells, transcription of *Opa1* increased likely involved in mitochondrial remodeling, including cristae shaping and serving as a regulator of cholesterol shuttling (41).

Living in LL, in addition to overall constant conditions, profoundly affects mitochondrial function in peri/pubertal and adult Leydig cells. The mtDNA decreases as well as *Cytc* (encoding subunit of respiratory protein), mitochondrial membranous potential, and ATP production. Since mitochondrial respiration produces around 80% of ATP in adult Leydig cells (22), decreased mitochondrial function significantly affects energy cell status. Decreased mitochondrial activity observed in Leydig cells from peripubertal and adult rats is supported by decreased *Opa1* and *Mfn2* with the potential to increase mitophagy due to increased *Pink1* suggesting unbalanced mitochondrial dynamics connected with lower steroidogenesis. Observed disturbed mitochondrial function in Leydig cells could arise as decreased LH signaling in LL condition, having a substantial effect on mitochondrial physiology (15).

However, living in LL is associated with cumulative impacts of the disturbed rhythmicity of many hormones important for reproductive function. Indeed, in LL the sustained increased levels of blood corticosterone were observed, reflecting chronic stress condition. Long-term glucocorticoid exposure becomes maladaptive, leading to a broad range of disorders, including metabolic syndrome and obesity (42). Our results pointed to decreased T/C ratio in the LL regime, indicating an altered behavior and reduced activity. The balance of testosterone as anabolic and corticosterone as a catabolic hormone could be used as a physiological stress biomarker. In humans, the increased T/C has been associated with aggression and social dominance (43). Anyway, it has long been recognized that increased corticosterone may reduce testosterone production by inhibiting steroidogenic enzyme expression and activity (44) in addition to Leydig cell apoptosis activation (45). Still, Leydig cells from juvenile and peripubertal rats could be protected from the adverse corticosterone effect due to the increased level of *Hsd11b1*. The HSD11B1 is a bi-directional oxidoreductase that inactivates biologically active glucocorticoid or activates inert metabolite into functional form and thus acts as a local regulator of glucocorticoid levels (29). However, in rat Leydig cells HSD11B1 changes from a primary reductase to predominant dehydrogenase during pubertal maturation (46). In adult Leydig cells from LL rats, the observed lower *Hsd11b1*expression may contribute to decreased testosterone production.

Additionally, melatonin is a principal darkness hormone with various physiological and metabolic functions, including influence on body weight, plasma insulin and leptin levels (47), and modulation of energetic metabolism (48). In rat males kept under LL conditions, low melatonin levels have been shown regardless of circadian time (49). Low blood melatonin is associated with decreased activity, increased visceral adiposity, and disturbed circadian rhythm and behavioral parameters. Interestingly, such effects, including melatonin secretion and activity pattern, were not observed in rats living in the continuous dark (49). Our previous results did not support direct melatonin effect on Leydig cells through melatonin receptors, but melatonin deprivation exerted a positive effect on steroidogenic and Leydig cells’ clock genes (4). In the present study, increased *Mntr1a* transcription was detected in Leydig cells from immature and peripubertal rats that lived in LL conditions.

In conclusion, the data showed that during Leydig cell differentiation, the increased mitochondrial biogenesis occurred together with the cells’ increased ability to produce testosterone. However, up-regulated mitochondrial biogenesis is not related to increased energetic cell capacity, suggesting dissociation of the mitochondria’s energetic and steroidogenic function during Leydig cell maturation. Growing up in a constant (LL) environment changed the circadian system and slowed down Leydig cells’ maturation by reducing the endocrine and energy capacity of cells, which led to a delay in reproductive development. Leydig cells responded to the free-run challenge by altered expression patterns of genes related to steroidogenesis, mitochondrial dynamics, and clock (**Figure 7B**), leading to unbalanced steroidogenesis, especially in the mitochondrial portion.

## Data Availability Statement

The datasets generated for this study are available on request to the corresponding author.

## Ethics Statement

The animal study was reviewed and approved by the Local Ethical Committee on Animal Care and Use of the National Council for animal welfare and the National Low for Animal Welfare (No. 323-07-0-08975/2019-05).

## Author Contributions

DM—acquisition of the data, analysis and interpretation of the data, revising manuscript critically for important intellectual content. MM—acquisition of the data, analysis and drafting the figure, revising manuscript critically for important intellectual content. AB—acquisition of the data, analysis and interpretation of the data. SA—acquisition of the data, analysis and interpretation of the data, revising manuscript critically for important intellectual content. TK—the conception and design of the research, acquisition of the data, analysis and interpretation of the data. drafting the manuscript. All authors contributed to the article and approved the submitted version.

## Funding

This research was supported by the Serbian Ministry of Education and Technological Development grant no. 173057 and CeRes grant, and the Autonomic Province of Vojvodina grants no. 3822 and no. 2130.

## Conflict of Interest

The authors declare that the research was conducted in the absence of any commercial or financial relationships that could be construed as a potential conflict of interest.
