# Supplementary figures and images for: Growing Up Under Constant Light: A Challenge to the Endocrine Function of the Leydig Cells
Source: Front Endocrinol (Lausanne). 2021 Mar 16;12:653602. doi: 10.3389/fendo.2021.653602 (PMC8008111; doi:10.3389/fendo.2021.653602)

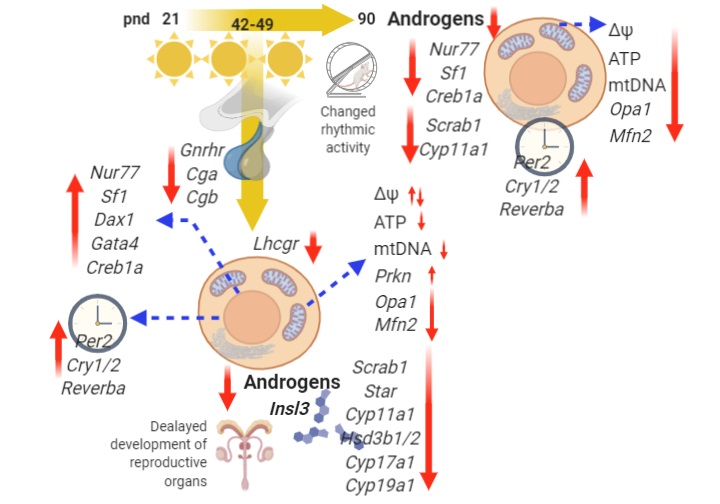

Supplement: Supplementary file 1 [file Image_1.jpeg]
