# Supplementary material for: Growing Up Under Constant Light: A Challenge to the Endocrine Function of the Leydig Cells
Source: Front Endocrinol (Lausanne). 2021 Mar 16;12:653602. doi: 10.3389/fendo.2021.653602 (PMC8008111; doi:10.3389/fendo.2021.653602)
Supplement: Supplementary file 2 [file Table_1.docx]

Supplemental Table 1. Primers Sequence

|  | **Access bank code** | **Primer sequence** |
| --- | --- | --- |
| ***Arr19*** | NM_001013142 | F:5'-TGCTGCAATCTCTTGTTTCC-3'  R:5'-TGTGCCATGATGAAAAAGGT-3' |
| ***B2m*** | NM_012512.2 | F: 5’-GCGTGGGACGAGCATCAGGG -3’  R: 5’-CTCATCACCACCCCGGGGACT-3’ |
| ***Bmal1*** | NM_024362.2 | F:5'-AAGAGGCGTCGGGACAAAAT-3'  R:5'- TTCCGGGACATCGCATTG-3' |
| ***Cga*** | NM_053918.2 | F:5'-CAGTGTATGGGCTGTTGCTTCT-3'  R:5'-GGAACCAACATTGTCTTCTTGGA-3' |
| ***Clock*** | NM_021856.1 | F:5'-ACAGCCCCACTGTACAATACGA-3'  R:5'- TGCGGCATACTGGATGGAAT-3' |
| ***Cox4i2*** | NM_053472 | F: 5'-CACAGCCCAGGAAGTGCTGCTA-3'  R: 5'-TGTGCAGTAAGGCTCATCCGGC-3' |
| ***Creb1a*** | NM_134443.1 | F:5'-CCAACCCCGATTTACCAAACT-3'  R:5'-CCATTGTTAGCCAGCTGTATTGC-3' |
| ***Cry1*** | NM_198750.2 | F:5'-ACCATCCGCTGCGTGTACAT-3' R:5'-AGCAAAAATCGCCACCTGTT-3' |
| ***Cry2*** | NM_133405.1 | F:5'-TTCCCAAGGCTTTTCAAGGA-3' R:5'-TCCCGTTCTTTCCCAAAGG-3' |
| ***Cyp11a1*** | NM_017286 | F: 5'-CAACATGGAGTCAGTTTACA-3'  R: 5'-GACCCTCGCAGGAGAAGAGA-3' |
| ***Cyp17a1*** | NM_012753 | F: 5'-GCCACGGGCGACAGAA-3'  R: 5'-GCCTTTGTTGGGAAAAATCG-3' |
| ***Cyp19a1*** | NM_017085 | F: 5'-TCCTCCTGATTCGGAATTGTG-3'  R: 5'-GGCCCGATTCCCAGACA-3' |
| ***CytC*** | NM_012839 | F:5'-GCAAGCATAAGACTGGACCAAA-3'  R:5'-TTGTTGGCATCTGTGTAAGAGAATC-3' |
| ***Dax1*** | NM_053317 | F: 5'-GCCGAGGGCCCCCTGGTGGGAC-3'  R: 5'-GATCTGGAAGCAAGGCAAGT-3' |
| ***Drp1*** | NM_053655.3 | F: 5’-AGGTTGCCCGTGACAAATGA-3’  R: 5’-CACAGGCATCAGCAAAGTCG-3’ |
| ***Fis1*** | NM_001105919.1 | F: 5’-ACGCCTGCCGTTACTTCTTC-3’  R: 5’-GCAACCCTGCAATCCTTCAC-3’ |
| ***Fshb*** | NM_001007597.2 | F: 5'-TAGCCAACTGCACAGGACAT-3'  R: 5'- CACAGCTATGGCAGCAGACT-3' |
| ***Gapdh*** | NM_017008 | F: 5'-TGCCAAGTATGATGACATCAAGAAG-3'  R: 5'-AGCCCAGGATGCCCTTTAGT-3' |
| ***Gata4*** | NM_144730 | F: 5'- GATGGGACAGGACACTACCTATGC-3'  R: 5'- GGCGCTGAGGCTTGATGA-3' |
| ***Gnrhr*** | NM_031038.3 | F: 5’-CTGTTCAGTGGTATGCTGGAGA-3’  R: 5’-AGTGGGTCACACATTGCGAG-3’ |
| ***Hsd11b1*** | NM_017080 | F: 5'-AAATACCTCCTCCCCGTCCT-3'  R: 5'-TTTCTCTTCCGATCCCTTTG-3' |
| ***Hsd11b2*** | NM_017080 | F: 5'-CAAACCCTTCCCCCACAG-3'  R: 5'-GGCTGGGCTTTTCTTAACAG-3' |
| ***Hsd17b4*** | NM_024392 | F:5'-CCTTTGGCTTTGCCATGAGA -3'  R:5'-CAATCCATCCTGCTCCAACCT -3' |
| ***Hsd3b1/2*** | NM_001042619.1 | F: 5'-GACAGGAGCAGGAGGGTTTGTGG-3'  R:5'-CTCCTTCTAACATTGTCACCTTGGCCT-3' |
| ***Insl3*** | NM_053680.1 | F:5’-CGCAGTGTGGCCACCAA-3’  R:5’-CCTGAGCCCTACAATCCTTCAG-3’ |
| ***Lhb*** | NM_012858.2 | F: 5'- TCTTCTGATGCCCACCCACTA -3'  R: 5'-TATTGGGAGGGATGGTTAGAACA-3' |
| ***Lhcgr*** | NM_012978 | F:5'-CGGGCTGGAGTCCATTCA-3'  R:5'-TTCTTTGGAGGGCAGTGTTTTC-3' |
| ***Mfn1*** | NM_138976.1 | F: 5'-CCTTGTACATCGATTCCTGGGTTC-3'  R: 5'-CCTGGGCTGCATTATCTGGTG-3' |
| ***Mfn2*** | NM_130894.4 | F: 5'-TCAAGCGCCAGTTTGTGGAG-3'  R: 5'-CACAGATGAGCAAATGTCCCAGA-3' |
| ***mtNd1*** | NC_001665.2:2740-3694 | F: 5'-GCGTGGGAGGAGCATCAGGG-3'  R: 5'-GCGAATGGTCCTGCGGCGT-3' |
| ***Mtnr1a*** | NM_053676.2 | F: 5′-GGAATACTGAACACGCACAAAGG -3′  R: 5′-GCTAAGTGTGGGCCCCTAAGA -3′ |
| ***Nr1d1 (Reverba)*** | NM_001113422.1 | F:5'-GAGCATCCAGCAGAACATCCA-3'  R:5'-TTGCGATTGATACGGACAATG-3' |
| ***Nr1d2 (Reverba)*** | NM_147210.1 | F:5'-GAACGAGAATTGCTCCATCATG-3' R:5'-CGACATTCCCACGGACAGA-3' |
| ***Nrf1*** | NM_001100708 | F: 5'-GACCATCCAGACGACGCAAGCA-3'  R: 5'-ATGGGCGGCAGCTTCACTGTT-3' |
| ***Nur77*** | NM_024388 | F:5'-GGCATGGTGAAGGAAGTTGT-3'  R:5'-GATTGGTAGGGGAGGCATCT -3' |
| ***Opa1*** | NM_133585.3 | F: 5'-AAAAGCCCTTCCCAGTTCAGA-3'  R: 5'-TACCCGCAGTGAAGAAATCCTT-3' |
| ***Per1*** | NM_001034125.1 | F:5'-CCTGCACACCCAGAAGGAA-3'  R:5'- GAGGTGTCAAGCCCACGAA-3' |
| ***Per2*** | NM_031678.1 | F:5'-GGAAGGAGGCCCAGACGTA-3'  R:5'- TGGGTCCATTTCGTTAGAAACA-3' |
| ***Pink1*** | NM_001106694.1 | F: 5’-CAAGCAAGTGTCTGACCCAC-3’  R: 5’-GCTTCATACACAGCGGCATT-3’ |
| ***Ppargc1a*** | NM_031347 | F: 5'-AGCCGTAGGCCCAGGTATGACA-3'  R: 5'-TGCTTGGCCCTTTCAGACTCCC-3' |
| ***Prkn*** | NM_020093.1 | F: 5’-CTTCCAGCTCAAGGAAGTGG-3’  R: 5’-CAGAGGCATTTGTTTCGTGA-3’ |
| ***Rorb*** | NM_001270958.1 | F:5'-CAGGAACCGTTGCCAACAC-3' R:5'-GGACATCCTCCCAAACTTCACA-3' |
| ***Scarb1*** | NM_031541.1 | F:5'-GAAGGCATCCCCACTTATCG-3'  R:5'-CGGACCCGTTGGCAAA-3' |
| ***Sf1*** | NM_001191099 | F:5'-CCGCGGGCATGGACTA-3'  R:5'-TTGTCACCACACACTGGACACA-3' |
| ***Star*** | NM_031558 | F: 5'-AGCCAGCAGGAGAATGGAGAT-3'  R: 5'-CACCTCCAGTCGGAACACCTT-3' |
| ***Tfam*** | NM_031326 | F: 5'-TATAGTCGTCGGCCCGAGGGAT-3'  R: 5'-AAGGCTGACAGGCGAGGGTATG-3' |
| ***Nr3c1*** | NC_005117.4 | F:5'-CGGTTAATCTGCACAGCCTAT -3'  R:5'-AAAATGGGTCGGTGCTTCTA -3' |
