# Supplementary material for: Growing Up Under Constant Light: A Challenge to the Endocrine Function of the Leydig Cells
Source: Front Endocrinol (Lausanne). 2021 Mar 16;12:653602. doi: 10.3389/fendo.2021.653602 (PMC8008111; doi:10.3389/fendo.2021.653602)
Supplement: Supplementary file 3 [file Table_2.docx]

**Supplemental table 2. Rhythm parameters in running wheel animal’s activity from control (L/D) and rats exposed to constant light regime (L/L) (period fitted to 24h).** Rats from P21-P90 were exposed to constant light (L1-L5) or 14 h light/10 h dark (C1-C5) regime. Running wheel activity was monitored in 30 days duration, from P60 to P90. Presented rhythm parameters were obtained by Cosinor method.

| **Group** | **p value** | **Animals** | **Mesor** | **Amplitude** | **Acrophase (ZT)** |
| --- | --- | --- | --- | --- | --- |
| ***L/L*** | 0.02785 | L1 | 2.84975 | 7.330 | 9 h 10 min |
|  | 0.00434 | L2 | 5.94282 | 11.663 | 8 h 30 min |
|  | <0.00001 | L3 | 30.78314 | 72.049 | 11 h 9 min |
|  | <0.00001 | L4 | 30.98488 | 95.563 | 7 h 19 min |
|  | 0.00011 | L5 | 14.70428 | 24.464 | 5 h 46 min |
| ***L/D*** | <0.00001 | C1 | 48.03048 | 70.192 | 17 h 47 min |
|  | <0.00001 | C2 | 69.00511 | 101.334 | 17 h 58 min |
|  | <0.00001 | C3 | 34.78521 | 49.068 | 18 h 30 min |
|  | <0.00001 | C4 | 7.44897 | 10.8925 | 17 h 56 min |
|  | <0.00001 | C5 | 35.50560 | 49.5222 | 17 h 42 min |

**ZT** is Zeitgeber time; **p** *value* - probability of outcome
